# “Are we ready for robots that care for us?” Attitudes and opinions of older adults toward socially assistive robots

**DOI:** 10.3389/fnagi.2015.00141

**Published:** 2015-07-23

**Authors:** Maribel Pino, Mélodie Boulay, François Jouen, Anne-Sophie Rigaud

**Affiliations:** ^1^Department of Geriatrics, Assistance Publique-Hôpitaux de Paris, Hôpital BrocaParis, France; ^2^Faculté de Médecine, Université Paris DescartesParis, France; ^3^Laboratoire Cognitions Humaine et Artificielle, Ecole Pratique des Hautes EtudesParis, France

**Keywords:** socially assistive robots, technology acceptance, older adults, Mild Cognitive Impairment, dementia

## Abstract

Socially Assistive Robots (SAR) may help improve care delivery at home for older adults with cognitive impairment and reduce the burden of informal caregivers. Examining the views of these stakeholders on SAR is fundamental in order to conceive acceptable and useful SAR for dementia care. This study investigated SAR acceptance among three groups of older adults living in the community: persons with Mild Cognitive Impairment, informal caregivers of persons with dementia, and healthy older adults. Different technology acceptance questions related to the robot and user characteristics, potential applications, feelings about technology, ethical issues, and barriers and facilitators for SAR adoption, were addressed in a mixed-method study. Participants (*n* = 25) completed a survey and took part in a focus group (*n* = 7). A functional robot prototype, a multimedia presentation, and some use-case scenarios provided a base for the discussion. Content analysis was carried out based on recorded material from focus groups. Results indicated that an accurate insight of influential factors for SAR acceptance could be gained by combining quantitative and qualitative methods. Participants acknowledged the potential benefits of SAR for supporting care at home for individuals with cognitive impairment. In all the three groups, intention to use SAR was found to be lower for the present time than that anticipated for the future. However, caregivers and persons with MCI had a higher perceived usefulness and intention to use SAR, at the present time, than healthy older adults, confirming that current needs are strongly related to technology acceptance and should influence SAR design. A key theme that emerged in this study was the importance of customizing SAR appearance, services, and social capabilities. Mismatch between needs and solutions offered by the robot, usability factors, and lack of experience with technology, were seen as the most important barriers for SAR adoption.

## Introduction

Increase in life expectancy and population aging has contributed to the rise of the number of elderly individuals living with an age-related disability or a chronic disease. In order to improve health outcomes, quality of life, and control the costs associated with health and social care in this age group, there is growing social and economic pressure to help older adults to live at home for as long as possible (Fujisawa and Colombo, 2009). However, cognitive disability can seriously compromise independent living in old age, particularly when it stems from progressive conditions, such as some forms of Mild Cognitive Impairment (MCI), Alzheimer's disease (AD) and related dementias. Persons with dementia often require a complex care approach combining medical, social and preventive services. With the progression of the disease, the help needed for the execution of daily tasks normally increases, leading to burden of informal caregivers, and in many cases to institutionalization (Alzheimer's Association, 2012). Despite the importance of efforts and policies to improve care delivery at home, living at home until the very end of life remains a promise, not a reality for many persons living with dementia. Dealing with this situation represents a current and future challenge for society that has been increasingly addressed by assistive technology.

Socially Assistive Robots (SAR) are an emerging form of assistive technology encompassing all robotic systems capable of providing assistance to the user by means of social interaction (Feil-Seifer and Mataric, 2005; Broekens et al., 2009; Flandorfer, 2012). SAR can deliver help at different levels (Rich and Sidner, 2009): (a) supporting user's cognitive or functional abilities (e.g., task reminding and monitoring, navigation aids); (b) offering the user opportunities to enhance social participation and psychological well-being (e.g., communication and social applications, telepresence, companionship); (c) providing remote and continuous monitoring of user's health status (e.g., blood pressure or fall detection sensors); and (d) coaching the user to facilitate the promotion of healthy behavior and achievement of health-related goals (e.g., improving nutrition. physical activity).

The therapeutic use of SAR in the context of dementia care has received increasing attention over the last decade as illustrated by a growing body of research in this area (Libin and Cohen-Mansfield, 2004; Robinson et al., 2013; Mordoch et al., 2013; Moyle et al., 2014). Most of these studies have focused on Paro (Shibata and Wada, 2010), a therapeutic animal-like robot modeled on a baby harp seal, mainly employed to encourage social behavior and/or alleviate stress among persons with dementia. In the broader context of mental health interventions, Rabbitt et al. (2015) have recently described a number of roles for SAR, including: a companion (e.g., SAR that work in an analogous way to trained therapy animals), a therapeutic play partner (e.g., SAR used to help children build clinically relevant skills), and a coach or instructor (e.g., SAR provide instruction, encouragement and supervision to users in activities such as weight loss or physical exercise).

SAR cover a wide range of design solutions: *machine-like robots*, which have an unequivocal mechanical and computer-like aspect; *human-like robots*, whose form resembles a human body and/or have human facial features (e.g., eyes, nose, mouth, eyelids, etc.); *androids* or very realistic human-like robots; *mechanical human-like robots* which combine human-like and machine features; *animal-like robots* that simulate animal behavior and morphology; and *mechanical animal-like robots* which combine animal-like and machine features. These categories were defined by DiSalvo et al. (2002), MacDorman and Ishiguro (2006), and Walters et al. (2009). Mobility is another common feature of these systems although it is not mandatory. Locomotion, when available, allows the robot to move around in a particular environment, follow or locate a user or an object either by being operated at distance or autonomously guided.

Studies conducted on SAR acceptance among elderly people have shown that several robot-related variables appear to positively influence technology acceptance and intention to use these systems, for instance: perceived usefulness (e.g., facilitating care delivery, enhancing safety at home) (Arras and Cerqui, 2005; Scopelliti et al., 2005; Boissy et al., 2007); perceived enjoyment (e.g., pleasure associated with its use); robot appearance (e.g., having a small size and friendly aspect); perceived sociability (e.g., robot being caring, empathic, intelligent, exhibiting human-like communication capabilities) (Dautenhahn et al., 2005; Broadbent et al., 2010; Heerink et al., 2010; Wu et al., 2012); perceived adaptivity (e.g., robot being controllable and having a predictable behavior) (Dautenhahn et al., 2005; Scopelliti et al., 2005).

Conversely, other robot-related factors have been identified as having a negative impact on SAR acceptance: lack of trust in the robot (e.g., safety concerns) (Scopelliti et al., 2005); robot conveying a negative representation of prospective users because of a stigmatizing aesthetic (e.g., presupposing the user is isolated, dependent, and/or frail) (Hirsch et al., 2000; Neven, 2010); space requirements for the robot (e.g., important size or mass of the system) (Scopelliti et al., 2005; Young et al., 2009); robot appearance (e.g., reluctance toward humanoid robots) (Arras and Cerqui, 2005; Dautenhahn et al., 2005; Wu et al., 2012); accessibility issues (e.g., technology perceived too complex, high costs) (Young et al., 2009); and ethical concerns (e.g., reduction of social contact, replacement of human presence) (Arras and Cerqui, 2005; Dautenhahn et al., 2005; Harmo et al., 2005; Scopelliti et al., 2005; Sparrow and Sparrow, 2006; Wu et al., 2012). The influence of individual factors such as age, gender, cognitive abilities, education level, technology experience, cultural background, on SAR acceptance has also been addressed in some studies (Scopelliti et al., 2005; Broadbent et al., 2010; Flandorfer, 2012).

**Figure 1:**
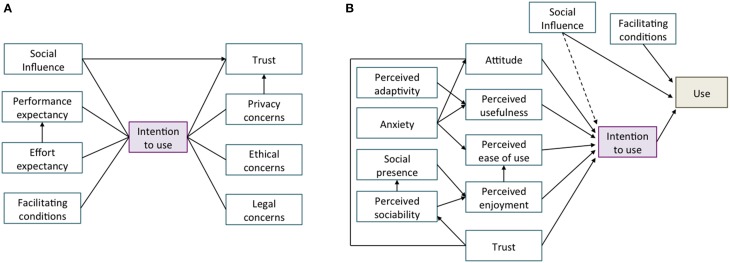
**Technology Acceptance Models applied to the context of SAR. (A)** Structural model of determinants of home healthcare robots adoption (Alaiad and Zhou, 2014); **(B)** The Almere model for assessing acceptance of assistive social agent technology by older adults (Heerink et al., 2010).

Many of these factors have been examined by different Technology Acceptance Models (TAM) developed to explore aspects that contribute, or hinder, the acceptance and use of robot technology. Such is the case of the Unified theory of acceptance and use of technology (UTAUT) model (Venkatesh et al., 2003) applied to the use of home healthcare robots (Alaiad and Zhou, 2014), or the Almere model (Heerink et al., 2010), also based on the UTAUT, suggested to assess the acceptance of assistive social agent technology by older adults. These models are built on the assumption “usage intentions” of the potential consumer regarding a particular technology are strongly correlated with subsequent use. In the field of assistive technology TAM may be valuable for guiding technology design because they allow understanding of variables that influence the acceptance of assistive devices. With this information, designers in the field of SAR may be able to conceive systems that are more likely to be adopted (Beer et al., 2011). Figure 1 presents determinants of technology acceptance and key moderators included in the two aforementioned models.

Concerning SAR, one of the factors that has been found to influence technology acceptance is the stakeholder category to which the potential user belongs: this means being either a patient, a professional or a person outside the healthcare environment (Broadbent et al., 2010; Alaiad and Zhou, 2014). Indeed, when used within the care context of a condition involving multiple care providers, such as dementia, SAR may serve different purposes depending whether the user is the person with cognitive impairment, a formal or an informal caregiver. Research in this field has shown that needs vary greatly among these stakeholder groups (Orrell et al., 2008; van der Roest et al., 2008). Therefore, it can be expected that specific needs in each stakeholder groups influence how these actors accept and adopt assistive technology (Topo et al., 2007; Topo, 2009). In the same line of ideas, it seems reasonable to expect that the severity of symptoms, or the stage of the disease, influences patients and caregivers' needs, and subsequently the intention to use SAR as any other support service. In this respect, Hawkey et al. (2005) noted that the interests of persons with dementia and those of informal caregivers might compete regarding both, a problematic situation and a potential solution. These authors conducted a needs-assessment study for an assistive technology device designed to store and automatically deliver information to address repetitive questioning in dementia. Results showed that some persons with dementia preferred to be given detailed information regarding un upcoming event a week or two ahead of time, whereas some caregivers preferred to limit the amount of information provided, and shorten the time to give this information, in order to avoid further repetitive questions (e.g., half hour or less before the event). The study confirmed the importance of interviewing both, the caregiver and the person with dementia, to have a good understanding of the impact of assistive technology on the dyad. Information collected from both sides should be useful to define systems requirements and increase its acceptability.

Most studies on SAR conducted in the dementia care context have only focused on one stakeholder group (For a review see Mordoch et al., 2013). Thus, little is known about how the views of persons with cognitive impairment and caregivers converge or diverge regarding the acceptance of SAR. A more comprehensive approach should include both groups' perspectives to better understand technology acceptance and usage intention of SAR in the general context of dementia care. In this study we seek to obtain data that will help to address this research gap.

The aim of this exploratory study was to clarify several aspects related to the acceptance of SAR by older adults. In particular, we were interested in examining if opinions and attitudes toward SAR differed among three groups of older adults living in the community: healthy elderly individuals, persons with MCI, and informal caregivers of persons with dementia. Different technology acceptance questions related to (a) robot features, (b) user characteristics, (c) potential applications, (d) feelings about technology, (e) ethical issues, and (f) facilitating or hindering factors for SAR acceptance, were addressed in a mixed-method study. The role of individual factors on SAR acceptance was examined as well (e.g., age, gender, education, health status, technology experience). Results from this study are expected to contribute to a better understanding of users' needs and system requirements for the development of SAR intended to support older adults with cognitive impairment at home and their informal caregivers.

## Materials and methods

### Participants

A total of 25 elderly individuals living in the Paris area (France) were enrolled in this study. Among the participants were 10 individuals with MCI, seven informal caregivers of persons with dementia, and eight healthy older adults (HOA). The group of HOA was included in order to contrast their opinions with those of participants confronted to cognitive impairment and/or dementia. Table 1 summarizes characteristics of the sample.

**Table 1:** **Summary of the sample characteristics**.

**Participants**	**MCI**	**Caregivers**	**HOA**	**All**
Number (female, male)	10 f(6), m (4)	7 f(5), m (2)	8 f(6), m (2)	25 f(17), m (8)
Mean age Range	71.5 65–83	68.2 858–81	77.75 69–86	72.6 58–86
Education level (*n*)	Elementary (0) Secondary (6) Higher (4)	Elementary (0) Secondary (1) Higher (6)	Elementary (1) Secondary (3) Higher (4)	Elementary (1) Secondary (10) Higher (14)
Volunteer work (*n*)	Yes (4) No (6)	Yes (4) No (3)	Yes (8)	Yes (16) No (9)
Health-status (0–12) (*SD*)	7.7 (4)	5.82 (1.79)	3.25 (2.37)	5.59 (3.06)
Technology use score (0–15) (*SD*)	10.5 (3.59)	11.28 (3.45)	11.12(3.04)	10.92(3.26)
Attitudes toward new technologies (0–6) (*SD*)	3.3 (1.88)	4.14 (1.67)	4.25(1.28)	3.84 (1.65)

*Volunteer work was rated “yes” for older adults who performed regular volunteer work for a church, charity or other community group and “no” for those who did not engage in this kind of activities, all the participants except for one caregiver were retired or had never been employed; Health status, number of health problems (0, excellent; 1–4, good; 5–8, fair; 9–12, poor); Technology use, number of current technologies used (0–5, scare; 6–10, moderate; 11–15, regular); Attitudes toward new technologies is a composite score built from two measurements: interest in new technologies (0–3) and reactions to new technology-related products or services (0–3); SD, Standard Deviation*.

Inclusion criteria for the MCI group were: being 65 years old or older, having received a clinical diagnosis of MCI according to the European Consortium on Alzheimer's Disease Working Group on MCI (Portet et al., 2006), living in the community, and not having any other medical condition or psychiatric disorder severe enough to preclude participation in the study. Cognitive status in this group was evaluated by the Folstein Mini-Mental State Examination (MMSE) (Folstein et al., 1975) and a battery of neuropsychological tests targeting memory, language, visuo-spatial capacities, and problem-solving skills. Persons with MCI also underwent a complete physical and neurological examination, including laboratory tests as well as cerebral imaging.

Inclusion criteria for the group of informal caregivers were: living in the community, being primary caregiver (spouse, adult children, other relative or friend) who cared, at least once a week, for an older adult who had been diagnosed with mild-to-moderate dementia, based on the Diagnostic and Statistical Manual of Mental Disorders (DSM IV) criteria (American Psychiatric Association, 1994). For HOA inclusion criteria were: being 65 years old or older, having a general preserved cognitive functioning, and living in the community. Caregivers and HOA with severe illnesses or psychiatric disorders were also excluded.

Participants in the MCI group and informal caregivers were recruited through the AP-HP Broca Memory Clinic (Paris). HOA were recruited through local senior associations in the Paris region. All participants volunteered for the study. The University Paris Descartes ethical committee approved the study protocol.

### Study design and data collection

A mixed-method approach, including a short self-administered questionnaire and a series of focus groups, was used for data collection and analysis. The questionnaire was structured in two sections: Part A covered socio-demographic information including age, gender, education, volunteering status, self-rated health status, use of current technologies, and interest in new technologies. Part B covered a number of domains assessing the global appreciation of SAR: (a) robot's appearance, (b) potential applications, (b) robot social ability, (d) perceived usefulness, and (e) current and future intention to use (See Supplementary Material, Appendix 1). These factors were selected based on constructs included in the Almere model (Heerink et al., 2010) and previous studies on SAR acceptance (Beer et al., 2011; Flandorfer, 2012). Content and usability of the instrument were first tested and refined through a pilot assessment among seven healthcare professionals working in the area of assistive technology for dementia care. Based on their feedback some items and the general structure of the questionnaire were reviewed in order to keep it easy to understand and complete. A booklet containing the pictures and descriptions of different SAR was distributed with the questionnaire as support material (See Supplementary Material, Appendix 2).

Participants were allocated to one of the seven focus groups that were purposefully heterogeneous (i.e., MCI, informal caregivers of persons with dementia, and HOA) (Table 2). Focus groups were digitally recorded, fully transcribed and subjected to content analysis using principles described by Strauss and Corbin (1998). Dedoose version 4.3.87 (Dedoose, 2012), a web application for mixed methods research, was used for qualitative data analysis. Data from the questionnaires were analyzed using descriptive and non-parametric statistical techniques and R statistical package version 2.13.2 (R Development Core Team, 2011).

**Table 2:** **Focus groups composition**.

**Focus group**	***n***	**Mean age (range)**	**Group**	**Gender composition (female, male)**
1	3	72.6 (65–83)	MCI	f (3)
2	3	73 (65–81)	Caregivers	f (3)
3	3	72.66 (65–81)	MCI	f (1), m (2)
4	4	79.25 (69–86)	HOA	f (3), m (1)
5	4	64.75 (58–72)	Caregivers	f (2), m (2)
6	4	76.25 (69–86)	HOA	f (3), m (1)
7	4	69.75 (68–73)	MCI	f (2) m (2)

### Material

**Figure 2:**
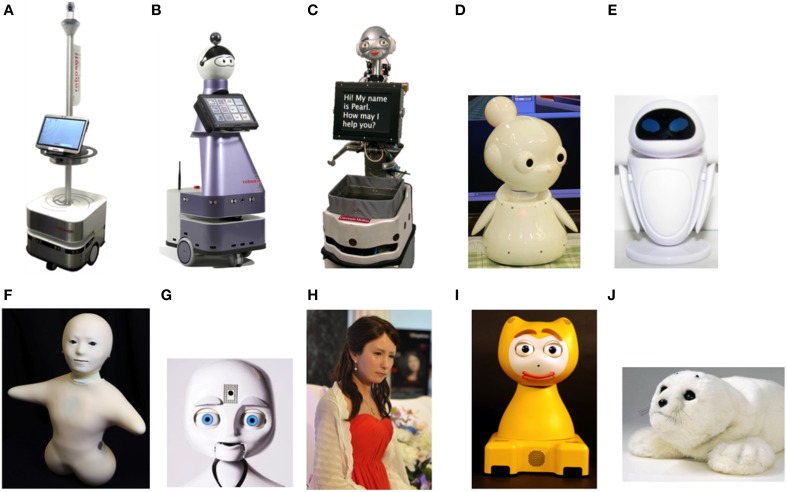
**Robots presented in the focus groups and design category**. *Machine-like:* **(A)** RobuLAB 10; *Mechanical human-like:* **(B)** Kompaï, **(C)** Pearl, **(D)** Mamoru-kun (little protector), **(E)** Eve (from Wall-E a Pixar film); *Human-like:* **(F)** Telenoïd, **(G)** Nexi; *Android:* **(H)** Geminoid F; *Mechanical animal-like:* **(I)** iCat: *Animal-like:* **(J)** Paro.

The RobuLAB 10 robot (Figure 2A) was used for a live demonstration of the robot. RobuLAB 10 is a mobile platform intended to provide cognitive and social support to older adults. Robot input devices include a voice-based control system and a touch-screen. For high-level control and user interfaces, the robot uses a Tablet PC with a 12.1” Premium WXGA (1280 × 800) display running Windows 7 (Dupourqué, 2009).

Support material for the focus group included a PowerPoint presentation with pictures and public available videos from different SAR projects that were projected during the discussion (Figure 2). The presentation covered a range of design solutions for SAR: machine-like, mechanical human-like, human-like, androids, mechanical animal-like, and animal-like. Other material included a video projector, a screen, a computer, and a video camera.

### Procedure

Potential participants were contacted by telephone and given information about the purpose and nature of the study. If interested, they were scheduled to participate in a focus group. The day of the meeting, participants read and signed an informed consent form prior to the beginning of the discussion. The meeting began with the introduction of the participants and a summary of the procedures that will be followed. Participants received the booklet with the robots' description and the questionnaire. They were then asked to complete Part A of the survey related to demographic information.

Two trained moderators led the focus groups. One of them made a live demonstration with the RobuLAB 10 and presented the scenarios. The second moderator conducted the discussion, raised questions and kept the conversation on the subject ensuring that all participants expressed their views. Sessions lasted between 1.5 and 2 h. The focus groups had a semi-structured format involving four sections:

1. *Demonstration of predetermined applications:* The demonstration robot was brought to the room using remote-controlled navigation. General robot's features, such as size, autonomy, weight, and interaction modalities (i.e., touchscreen and voice command), were presented. Seven short use-case scenarios, describing the interactions between a fictional character (Mr A, 81 years old, living alone, memory complaints) and the robot, were presented to illustrate a number of tasks that may nowadays be accomplished by SAR. The presentation did not restrict potential usages of SAR to dementia care in order to avoid excluding HOA to represent themselves as potential users. Participants were invited to give their views on these SAR scenarios:
  - *Communication and social support:* using the e-mail or the video-call applications of the robot, Mr A can communicate with health professionals, distant caregivers, relatives and friends.
  - *Compensate cognitive impairment:* to compensate Mr A's memory problems the robot can look around for him in his home and remind him of specific events (e.g., drug intake, appointments, finding lost items).
  - *Affective computing applications:* by using sensors and algorithms, the robot can gather information about Mr A's emotional state. The robot can also exhibit emotional responses to enrich the interaction with the user.
  - *Detection of emergency situations:* by using sensors and algorithms, the robot is able to detect emergency situations and alert Mr A's caregiver and/or health professionals (e.g., fall detection).
  - *Health monitoring:* by using sensors and algorithms, the robot can monitor and analyse Mr A's physiological signs or behavioral patterns (e.g., sleep patterns, physical activity) alerting healthcare services in the case of atypical activity.
  - *Cognitively stimulating and entertainment applications:* the robot includes a number of applications allowing Mr A to take part in stimulating activities alone or in a group (e.g., electronic games, online courses, virtual tourism).
  - *Support for daily tasks:* through different applications the robot can assist Mr A with daily activities (e.g., journey planning, weather forecast, online grocery shopping).
2. *Discussion on potential applications for SAR:* participants were encouraged to give examples of problematic situations they face in their everyday life. Regarding caregivers, challenging situations evoked should involve the person they care for or their caregiver role. People were asked to imagine possible applications of SAR to make it easier to care for themselves, or for a relative in the caregivers group, and in a general way to improve their quality of life.
3. *Discussion on SAR design:* the moderator gave a brief introduction about different design solutions of SAR. Participants were asked to express their perceptions and opinions for 10 robots, described in Section Material Corresponding pictures and videos were projected on a screen. Other topic brought up in the discussion was the match between a robot's appearance and its functions.
4. *Discussion on conditions for the adoption of SAR:* in the last part of the meeting, individual, societal and ethical issues that could be considered to facilitate or hinder SAR adoption were addressed. Participants were invited to express anything that they thought was important and that was not discussed throughout the session. Finally, they were asked to complete the Part B of the questionnaire on general appreciation of SAR, including robot's design, perceived usefulness and intention to use.

## Results

### Questionnaires

#### Preferences regarding SAR design

**Figure 3:**
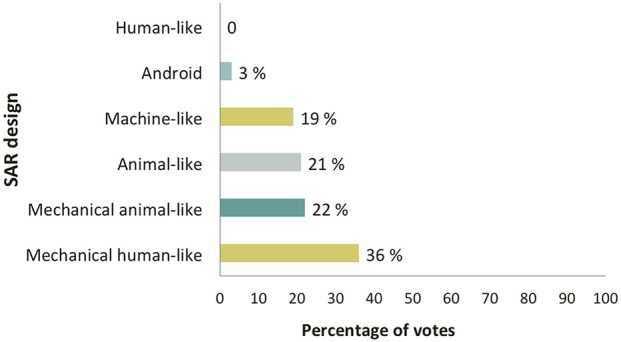
**Preferences regarding SAR design**.

As far as the general design of the robot was concerned, most participants preferred a mechanical human-like robot integrating some anthropomorphic facial features within a global mechanical-looking design. Mechanical animal-like, animal-like and machine-like robots received a similar percentage of votes. Android robots received only a few votes, and human-like robots did not get any at all (Figure 3). Regarding group preferences, participants in the HOA group rated highest the machine-like design, caregivers preferred the mechanical human-like design, and persons with MCI preferred the animal-like design.

Overall results showed that the degree of human likeness in the robot design was not considered a fundamental feature. Concerning the facial expressions of the robot, results showed that HOA were little interested in a design with realistic human-like features (0.75/3), while participants in the MCI and caregivers group were moderately interested in this kind of design (1.22/3 and 1.86/3, respectively). The representation of emotional capabilities through facial expressions obtained a moderate score in the MCI and caregivers groups (1.4/3 and 1.5/3, respectively), whereas HOA were less enthusiastic about this aspect (0.86/3).

#### Services and functionalities

**Figure 4:**
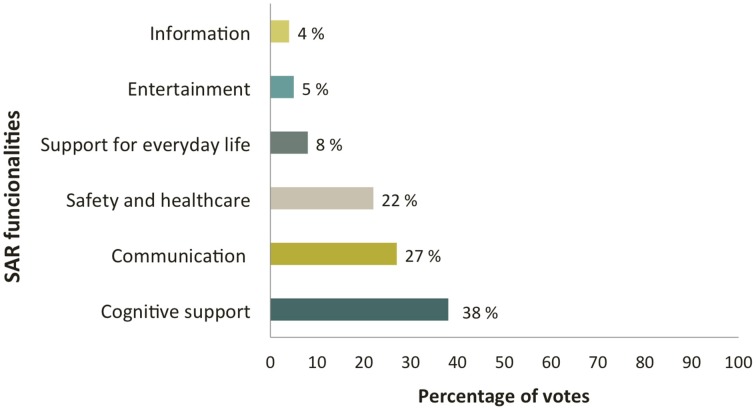
**Preferred functionalities for SAR**.

The most preferred functionalities for SAR, when considering all the participants, were: (a) cognitive support applications to compensate cognitive impairment (e.g., locating lost items, task reminding); (b) communication services to keep an active social life (e.g., video calls, email); (c) risk prevention and healthcare applications (e.g., falls detection, management of critical situations), and (d) applications for supporting everyday tasks (e.g., online grocery shopping, journey planning, simplified Internet access) (Figure 4). Other functionalities mentioned were entertainment (e.g., music, poetry, and reading) and information and news applications, for keeping the user up to date with current events (e.g., broadcast news sources). In the group of informal caregivers, it was suggested to develop a “life memory album” available via the robot, to support autobiographic memory in persons with memory loss and encourage communication with caregivers and/or family members. This application could include multimedia material, such as a genealogical tree, pictures and/or videos of significant moments of the life of the person. Regarding differences between groups, HOA rated preferred functionalities for SAR in order of preference: (a) communication and social support, (b) entertainment, (c) information. In the caregivers group applications were rated as follows: (a) safety and healthcare for care recipient, (b) compensation for cognitive impairment, (c) communication and social support. Finally, in the MCI group the rating observed was: (a) compensation for cognitive impairment, (b) communication and social support, (c) safety and health care.

#### Perceived usefulness and intention to use

**Figure 5:**
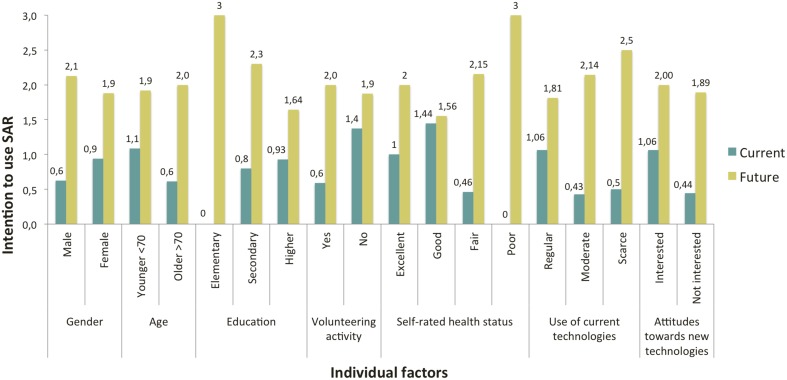
**Current and future intention to use SAR analyzed by individual factors**.

Concerning the intention to use the robot, results revealed that participants were more ready to use SAR in the future (*M* = 1.96. *SD* = 0.88), than at the present time (*M* = 0.84. *SD* = 0.98). This difference between current and future acceptance scores was observed in all user subgroups regardless of the variable used as a distribution factor (Figure 5). A Wilcoxon Signed-ranks test indicated that this difference was significant (*z* = −3.08. *p* < 0.002, two-tailed test).

Participants with MCI and caregivers had a more positive perception of the usefulness of SAR than HOA. Regarding the intention to use the robot, participants in the MCI and caregivers groups were more likely to accept to use the robot at the present time than HOA, although these scores were rather low in all groups, since they did not reach the average score of 1.5 of 3.0 (Table 3). Future intention to use was positively rated in all the three groups. However caregivers expressed less interest in using the system in the future compared to participants in the two other groups. A series of Fisher's exact tests were performed to examine the difference between groups regarding perceived usefulness, current and future intention to use, but no statistically significant difference was observed.

**Table 3:** **Perceived usefulness, current and future intention to use SAR**.

**Attitudes toward SAR**	**MCI (0–3) (*SD*)**	**Caregivers (0–3) (*SD*)**	**HOA (0–3) (*SD*)**	***F*-test *p*-value**
Perceived usefulness	1.9 (1.1)	1.86 (0.9)	1.13 (1.13)	0.41
Current intention to use	1.1 (0.99)	1.29 (1.11)	0.13 (0.33)	0.20
Future intention to use	2.2 (0.63)	1.67 (1.21)	2.13 (0.64)	0.29

With respect to the difference between current and future intention to use SAR, within each group, a series of Wilcoxon signed-rank tests were carried out. This difference was significant in the MCI (*W* = −36, *p* < 0.02, two-tailed test) and the HOA group (*W* = −36, *p* < 0.02, two-tailed test) but not for the caregivers group (*W* = −4, *p* > 0.05, two-tailed test). Some of the arguments given by participants in the different groups to explain their position regarding perceived usefulness and usage intentions for SAR are presented in Table 4. Finally, a summary of findings by group for each criterion assessed in the questionnaire is presented in Table 5.

**Table 4:** **Factors explaining usage intention for SAR among groups**.

**MCI**	**Caregivers**	**HOA**
**ARGUMENTS FOR**		
**Provides global support** “*It will take care of my needs”* “*It could be like a personal assistant”* **Provides companionship** “*It will be an amusing companion”* “*I will not feel lonely”* **Supports independent living** “*It will delay my entry into a retirement home”* “*It will allow me to continue to do my errands if I can not leave my home”* **Supports social life** “*It will help me to have social contact outside of my home”* **Supports safety at home** “*It will ensure my domestic security”*	**Provides global support** “*It can help the patient and his/her entourage”* **Supports social life** “*It could help my wife to have social contact outside of my home”* “*I'm really interested in having a robot like this because I think it could influence our relationship (with the care-recipient) in a positive way”* **Supports safety at home and alleviate caregiver's stress** “*The robot would contribute to alleviate my stress, when my husband will not longer be able to stay alone at home and I have to go out”*	**Provides cognitive stimulation** “*The robot could allow me to exercise my brain”* **Useful for other people** “*A robot may be useful for disabled people”* “*I'd have loved that my mother, who had dementia, had it”* “*Perhaps, in the future I will be needing some help”* “*When I'll be older, it could allow me to maintain my autonomy for as long as possible”*
**ARGUMENTS AGAINST**		
**Negative effect on autonomy** “*I believe the use of the robot will restrict my autonomy”* **Size of the robot** “*It could interest me after reducing its size”* **Privacy concerns** “*The idea of surveillance does not appeal to me*” **Fear of robots replacing humans** “*With robots like those, pretty soon real people won't be needed anymore. Robots will take the place of teachers, of everyone”*	**Suitability for persons with dementia** “*It seems difficult to adopt it because my relative is seriously affected by dementia”* “*This robot will be difficult to adopt because my relative does not have the capacity to adapt to new things”* “*At the current state of my wife's illness, we are not yet concerned”* **Negative attitudes toward technology** “*This type of robot will be a total stranger for my relative”* “*My relative is hostile to this type of technology”*	**Generational gap** “*This robot addresses a younger generation that is more familiar with new technologies”* **Perceived usefulness** “*Not useful to me because I am too young”* “*Not useful to me because I am still active”* **Superfluous** “*I already have a computer which gives me access to the same services”* **Difficulty to project oneself into the future** “*I prefer to avoid the question. I am afraid of what is coming next [in life]”* “*It is difficult to know in which state I will be in in the future, to estimate its usefulness”*

**Table 5:** **Summary of findings by group**.

**Criteria**	**MCI**	**Caregivers**	**HOA**
**Preferred design**	Animal-like design	Mechanical human-like design	Machine-like design
**Interest in human-likeness of robot's design**	Moderate	Moderate	Low
**Representation of emotional capabilities through SAR expressions**	Moderate	Moderate	Low
**Preferred applications**	1) Compensate cognitive impairment 2) Communication and social support 3) Safety and health care	1) Safety and healthcare for care recipient 2) Compensate cognitive impairment 3) Communication and social support	1) Communication and social support 2) Entertainment 3) Information/ news
**Perceived usefulness**	Moderate	Moderate	Low
**Current intention to use**	Low	Low	Very low
**Future intention to use**	Moderate-High	Moderate	Moderate-high

### Focus groups

The content of the discussions was transcribed and assigned to the 25 participants, taking into account the individual characteristics as descriptors (e.g., age group, gender, technology experience). An open coding system was used to identify, analyse, and categorize excerpts (i.e., relevant segments of speech) into parent codes (i.e., major themes), then into sub-codes, referring to secondary topics within major themes. Categories were compared with published literature on SAR acceptance (Heerink et al., 2010; Flandorfer, 2012). Two researchers conducted coding and differences were discussed until an agreement was reached. Throughout the coding process six parent-codes and 27 sub-codes were defined. A total of 373 excerpts were extracted from focus groups and assigned at least one of these codes. Parent codes, sub-codes and the number of excerpts and transcripts (i.e., each participant's discourse) associated to each theme are summarized in Table 6.

**Table 6:** **Factors explaining SAR acceptance among groups**.

**Parent code**	**Excerpts *n* = 373**	**Transcripts *n* = 25**	**Sub-codes**
Robot characteristics	159	25	Robot design
			Usability issues and accessibility
			Customization/ personalization
			Interaction modalities/ robot control methods
User characteristics	83	21	Cognitive and physical limitations
			Preferences and habits
			Technology experience
			Social and psychological needs
Potential applications	132	25	Cognitive support
			Communication and social life
			Robotic companionship
			Entertainment
			Risk prevention and healthcare
			Support for caregivers
Feelings about technology	199	25	Negative appreciation
			Positive appreciation
			Perceived usefulness
			Influence of media representations of robots
Ethical issues	68	20	Privacy
			Dignity
			Autonomy
			Vulnerability
			Risk of social isolation
			Fear of robots replacing humans
Facilitating conditions	15	18	Costs of the service
			Need of a supportive environment
			Promotion of intergenerational relationships

#### Content analysis and individual factors

Excerpts were coded using parent codes and sub-codes and tagged with relevant individual factors pertaining to each participant (e.g., gender, technology experience). For each specific theme percentages indicate the proportion of relevant occurrences found in a particular group (i.e., MCI, HOA, informal caregivers). Occurrences by group were previously normalized based on the relative number of cases. This section presents some key trends observed in the data and excerpts of verbatim illustrating relevant statements for each theme.

With regard to robot characteristics, personalization was a topic of much discussion (HOA 44.2%, MCI 33.4%, caregivers 22.4%) mainly with respect to the appearance of the robot, its behavior, and the choice of services: “*An interest thing could be to let people put whatever they want as the robot's head and that it has meaning for the person. Customization is important to make the robot more personal”* (MCI, 72 y/o). Customization was also considered a key aspect to make the robot usable by persons with physical disabilities: “*It is important to be able to set the height of the robot. You have to consider that there are tall people, small people, people who are seated, or bedridden”* (MCI, 64 y/o).

Caregivers were more concerned about usability issues (55.5%), including ergonomics, training, and support, than participants with MCI (16%) and HOA (28.5%). In general, most caregivers agreed that SAR involving computer interfaces would be inaccessible for persons with dementia and little technology experience: “*I think persons with dementia will be unable to use the robot. Somebody else would have to do it for him. Otherwise, training must be provided at the first stages of the disease. My husband now has difficulties using the telephone, even if he's used it for over 70 years. How can you expect him to learn to use an appliance that is completely new for him? This is completely utopian”* (Caregiver spouse, 72 y/o).

With respect to the robot form and appearance, strong negative opinions on giving a human appearance to the robot came from HOA (75%). In general, persons in this group considered that a robot was just a machine and should therefore have a machine-like appearance: “*I'm against humanoid robots. If you have a scientific mind, you may ask yourself, what is the purpose of this ventriloquist dummy anyway?*” (HOA, 79 y/o). Participants in the MCI and caregivers group had more positive opinions toward human-like robots. In these groups, a robot being capable of human-like communication was welcomed: “*If the robot is going to be part of my life it must be capable of communicating with me, being helpful is not enough”* (MCI, 83 y/o). The use of anthropomorphic forms by robot designers was appreciated to a certain degree, but a too realistic human appearance was considered problematic. For instance, caregivers argued that hyper-realistic representations could lead persons with dementia to confusion. These opinions also reflected the influence of media representations of robots and the field of robotics itself: “*There was this film [Blade runner] in which robots resembled humans so closely that they were confused with them. It was beautiful, but it was terrible at the same time”* (Caregiver children, 81 y/o). Participants in the MCI group claimed that they would have the feeling of being deceived: “*I don't like a humanoid robot because it gives you the illusion of being with someone and in reality you are still alone”* (MCI, 73 y/o).

User's characteristics were a matter of overall concern. Participants in the caregivers and MCI groups considered that user-friendly SAR could be particularly helpful to people with cognitive limitations (43.2 and 37.8%, respectively). These perceptions were in line with their opinions about the potential applications of SAR. Indeed, participants in both groups considered that SAR could provide cognitive support for everyday tasks (caregivers 46%, MCI 36.8%), for instance, helping the user to locate lost items, remind events and memories, or be oriented in time. There was also a positive perception of entertainment applications that could be implemented in the robot. However, a particular concern was raised about the conformity of these activities with the preferences and habits of the potential user: “*You say the robot could offer some electronic games. But you have to recognize that people who are in their eighties now are from a generation that is not used to play. They were taught to work, that's all. It seems difficult to ask people to do something that they have never done in their entire life”* (Caregiver spouse, 79 y/o).

Caregivers globally agreed that SAR could support them in their caregiving duties and alleviate their burden (87.5%). Participants in the HOA considered that SAR would be particularly helpful to support people with physical limitations (73.3%), or sensory impairment (71.4%). These opinions were consistent with the fact that in a general way HOA did not perceived the robot as being useful for themselves at the present time. Potential applications mentioned by HOA were also in agreement with the representation they had of prospective users of SAR (e.g., being frail, disabled, or isolated): “*I've met many young people, who are in their forties or fifties, who would be interested in a robot like this because they are paralyzed. People who have multiple sclerosis would be much more interested in it than an older person who has no experience with technology”* (HOA, f, 79). Most preferred functionalities for SAR in the HOA group were risk prevention and healthcare (55.6%), support for caregivers (55.6%), and communication and social life services (54.4%).

Robotic companionship was considered an interesting feature for participants in the MCI group (39.5%) followed by caregivers (32.3%). Nevertheless, it must be specified that his idea was found attractive as long as the primary goal of SAR was not to replace human contact: “*For some people it can be more pleasant to be with a robot than to be alone. It would also allow the caregiver to have some time away from the patient. But its use should not be generalized. For a person that still has a social life, seeing a human face is better than looking at a screen”* (Caregiver spouse, 65 y/o). Persons with MCI tended to perceive a robot companion as a distraction, a confidant, and a company for lonely people: “*This robot could be like a friend. The person wouldn't have the impression of being completely alone…24 hours it's a long time when you're alone”* (MCI, 73 y/o). Participants in the HOA group who agreed on the interest of the robotic companionship function (28.2%) estimated that it could be helpful for isolated people or for those with depression, but they saw no benefit of this feature for themselves.

Participants in all groups discussed ethical issues associated to the use of SAR. Also, independently from the group to which they belong, those who reported a higher perceived usefulness of SAR, or being ready to adopt a robot at the present time, discussed ethical aspects to a greater extent (37.5 and 41.8%, respectively) than participants who reported no perceived usefulness (26%) or current intention to use a robot (24.4%). However, only participants in the MCI group expressed their concern about the stigmatization that could result from the use of SAR: “*Some work has to be done if you don't want people to think that if they are given a robot it's because they are not worth a human company. People should think that the robot is there to help. There must be a way to present it in a positive way*” (MCI, 70 y/o).

MCI participants were more sensitive to privacy issues (56%) than caregivers (16%) and HOA (28%). A common view in the MCI group was that surveillance applications could be a threat to their privacy: “*We can not accept to use a robot for surveillance purposes. It is awful to that to someone who has been free and independent during all his life. Human freedom is a wonderful thing, and we must keep it during our whole life”* (MCI, 68 y/o). Nevertheless, individuals with MCI did express their interest in services that could contribute to their safety (49.1%), as long as these services did not involve video data gathering, for instance fall detectors and emergency call systems. Caregivers had a more positive perception of these risk-prevention applications for their potential to improve safety at home: “*It may be intrusive but, at the same time, integrating a security camera in the robot could be useful. My mother is alone at home during the night. If there was a camera, I could check from time to time if everything is OK. Between privacy and safety, it is not better to give priority to safety?”* (Caregiver children, 58 y/o).

Concerns about dignity were mainly pointed out by HOA participants (47.9%). These apprehensions were mostly associated to the appearance of the robot, particularly to the use of human or animal-like robots: “*It is quite worrying. We're giving elderly people machine companions. It is undoubtedly much better to have human companionship. Perhaps in some cases there is not choice, and that's sad”* (Caregiver spouse, 65 y/o). Participants who expressed their concerns about infantilizing elderly people with SAR also indicated no perceived usefulness of SAR (70.2%), and no current (67.1%) or future intention (83.6%) to use them. A feeling of mistrust toward some applications of the robot was reported, especially among caregivers (44.4%) and participants with MCI (36.2%). These apprehensions were related to the following aspects: the effectiveness of the robot, the replacement of human caregivers by robots, and unemployment that could result from it, data confidentiality, and safe use of the system. Finally, of particular concern among participants with MCI and caregivers was SAR costs being prohibitively high (46.8 and 38.7%, respectively), factor that could hinder their acquisition.

## Discussion

This study investigated SAR acceptance among three groups of older adults living in the community: healthy older adults, persons with Mild Cognitive Impairment, and caregivers of persons living with dementia. In this section, findings are discussed with respect to the main factors that were identified as having an influence on SAR acceptance, in particular those associated to group characteristics.

### Personalization: being able to adapt SAR to users' needs, preferences, and capabilities

One of the key themes that emerged in this study was the influence of personalization on SAR acceptance. Personalization may concern the general design of the robot (e.g., appearance, voice, gender), its behavior, the services it offers, or its social capabilities. These findings further support the idea of “customization needs,” which has been previously addressed in the literature on assistive robotics for elderly people. For instance, Meng and Lee (2006) emphasized that considering the heterogeneity of older people, a successful robot design must give priority to the user's preferences and the accurate understanding of individual needs, technological issues being of secondary importance. Sharkey and Sharkey (2010) in their study on ethical issues and robot care in old age, also concluded that customization would be the best way to develop robots that contribute to the well-being of users without restricting their individual rights. Findings from our study revealed as well that a wide range of heterogeneous needs should be taken into consideration when designing SAR to be used in the context of dementia care. Based on the opinions gathered in this study this need for personalization may be analyzed at two levels:

(1) *Group-related interests:* each participant in this study belonged to a group sharing a role (e.g., being caregiver of a person with dementia), a health-status (e.g., experiencing memory loss, being in good health), or similar self-representations (e.g., being an independent and active person). The demand for personalized services partly responded to the search for solutions that meet the needs resulting from a particular circumstance: caring for a dependent person, experiencing cognitive decline, having some physical limitations, or feeling isolated. Consequently, there was a rather large heterogeneity among the three groups regarding the services expected from a robot.

Participants in the caregivers and MCI groups sought to identify solutions that could help them deal with the daily problems they faced. Participants with MCI focused on cognitive and functional support services intended to improve their autonomy. These findings are consistent with those of Gross et al. (2011) on how persons with MCI had a positive view on SAR functions related to cognitive support in daily life (e.g., cognitive stimulation, items locator, event reminder). For their part, caregivers expressed their interest in applications that could contribute to improve the living environment of persons with dementia (e.g., safety at home) and make it easier to care for them (e.g., cognitive support). Therefore, they perceived the robot as a tool for stimulating and supporting the person they cared for, as an extra assistant, or a potential mediator between them and their loved ones.

Our findings are in line with previous studies that have investigated needs of informal caregivers of persons with dementia that may be met by assistive technology (Topo, 2009). These needs include: improving safety at home, reducing caregiver's stress and burden, having access to stimulating and meaningful activities adapted to people with dementia, dealing with social withdrawal and apathy of care recipients. However, it is worth noting that in our study the severity of dementia symptoms appeared to be a modulating factor for the acceptance of SAR among informal caregivers. In accordance with the present results, Frennert et al. (2013) observed that some caregivers considered that SAR could be useful for other older people but not for their relative, who would not be cognitively able to use a robot.

Finally, participants in the HOA group did not ask for personalization of SAR, basically because they did not identify themselves as potential users of these systems. Even if in our presentation we did not put the accent on the use of SAR for supporting older persons with cognitive or physical impairment, HOA who took part in this study naturally considered that SAR should target isolated frail elderly or disabled people rather than healthy active people. These results confirm findings from previous studies in which healthy elderly persons have expressed their unwillingness to imagine having an assistive robot (Neven, 2010; Wu et al., 2012; Frennert et al., 2013). HOA considered priority services for a robot those that could meet the needs of people with various disabilities (e.g., compensation for disabilities, health monitoring, aid for mobility) and personalization requirements, when expressed, were formulated in this direction.

There are similarities between opinions of HOA in our research and those described by Neven (2010) in his study about the representations that elderly people and robot designers have of prospective users of SAR. This author found that for some older adults who took part in his research, having or needing a robot was a signifier of old age, loneliness, and physical and/or cognitive deterioration. Furthermore, he analyzed how these individuals dissociated themselves from the representation they had of prospective SAR users by presenting themselves as healthy, active, and independent persons, who were helping the “others” by taking part in research. It is possible that in our study, negative representations that HOA had of prospective users of SAR would have also led them to distance themselves from the group of potential users.

The identity-signaling approach to divergence proposed by Berger and Heath (2008) may prove helpful to interpret this finding. This approach claims that one of the reasons why people diverge from others, for example with regard to cultural tastes or practices, is to make sure that their identity is correctly recognized and avoid misidentification (e.g., being associated to low-status or disliked others). If we acknowledge that SAR convey a symbolic meaning, it is important to examine the connotations related to their use. In our study, the meanings that HOA attributed to prospective users of SAR were rather negative. It is therefore understandable that they have firmly avoided to be considered part of this group.

The analysis of ageism conducted by Nelson (2005), suggesting that for some persons, having a negative perception of elderly people is a way of denying the self-threatening aspects associated with old age (e.g., becoming frail, dependent, isolated) and reducing the anxiety associated with the idea of aging, offers an alternative interpretation for our findings. Within the field of SAR, Frennert et al. (2013) discussed how the refusal observed among older adults to imagine having an assistive robot at home could be explained, in a similar way, to their reluctance to accept physical and cognitive changes related to aging.

(2) *Individual preferences and self-representations:* Most participants agreed on the importance of being able to configure the robot according to their preferences to make it more personal. The need for personalization primarily concerned the robot appearance, its name, gender, personality, voice, interaction modalities, and the choice of services. Although most participants felt that physical attributes of the robot were a secondary aspect with respect to functionality, the frequency with which robot appearance issues were raised allowed us to conclude that physicality might have a strong influence on SAR acceptance.

In our study, some robot designs caused rejection because they were associated with negative representations of aging or unethical care practices (e.g., stigmatization, deceptiveness). These findings confirmed that ethical issues described by Sharkey and Sharkey (2010), and Sparrow and Sparrow (2006), such as the risk of infantilization (i.e., disempowering effect associated with the conception of elderly with dementia reverting back to childhood) and deception (i.e., being induced to believe that robots are something that they are not) are issues of concern for older adults. On the contrary, several participants argued that appearance could have a positive influence on robot acceptance if it conveys pleasure, competence, and friendliness. Most of these positive features were related to the social capabilities of the robot and will be discussed later.

Results from this study suggests that it is important to allow potential users to customize robot's appearance because negative judgments about its design may affect compliance and be a reason for technology rejection. On the contrary, a “positive design” could improve technology acceptance, attachment to the system, and make the integration of the robot into the home environment easier. In this sense we could expect positive effects of personalization of SAR similar to those observed for mobile phones (Blom and Monk, 2003; Cui et al., 2007; Ho and Lee, 2011), PCs, or domestic vacuuming robots (Sung et al., 2009). In this respect, Broadbent et al. (2009) have pointed out that allowing the user to personalize the robot would help not only to accommodate individual differences but also to give users a sense of autonomy and control over the robot. Finally, it seems surprising that TAM in the field of SAR have not given enough attention to the influence of robot appearance on the acceptance and use of SAR. Research in this area should explore if robot design could be considered as a determinant of SAR acceptance.

### Beyond appearance and functionality, usage intention is linked to robot's social ability

Caregivers and participants with MCI agreed about the fact that the robot should not only be useful, but also pleasant and fun to use. These findings are consistent with those of Heerink et al. (2010) who observed that there was a strong correlation between “perceived enjoyment” and “intention to use” when assessing interactive robots among elderly users. In the same line of reasoning, Young et al. (2009) indicated that satisfying users' need for fun and entertainment increased the acceptance of SAR.

However, as pointed out by Heerink et al. (2006) one of the challenges of SAR design is to reach a balance between functionality, resulting from the technical configuration, and enjoyment, supported by the physical and “psychological” attributes of the robot (e.g., appearance, voice, social capabilities, personality). These authors have suggested that SAR acceptance combines both, a practical and a social dimension. The first refers to the perceived usefulness of the system, and the second to the willingness of end-users to engage in a social interaction with a robot. Indeed, potential users of SAR need to have a clear understanding of the practical gains that result from the use of these systems. The way perceived usefulness influences intention to use a technology, and subsequently, predicts its use, has been validated by different Technology Acceptance Models (Venkatesh et al., 2003).

The social dimension of technology acceptance is a complex factor since it involves several aspects that go beyond utility and design. In the present study, most participants in the MCI and caregivers group had a positive view of the social capabilities of the robot. Participants who were interested in robotic companionship also considered that some robot's features (e.g., human-like voice, subtle anthropomorphic traits, having a caring and empathic personality) could facilitate social human-robot interaction. These findings are in line with the view of Young et al. (2009) of successful domestic robotic interfaces as being somewhere in between a mechanical and a human-like appearance.

The higher perceived usefulness of robot social features (e.g., facial expressions) observed in the caregivers and MCI groups, compared to that observed in the HOA group, could be explained as follows. Caregivers are daily confronted with cognitive and psychological symptoms of dementia, such as apathy, social withdrawal, gradual loss of verbal communication abilities, or depressed mood. Therefore, it is understandable that they consider SAR as a potential tool to stimulate the person they cared for. In this scenario the robot social features would be a positive attribute. Also, we should consider that the highest acceptance of robotic companionship observed in our study was in the group of persons with MCI. Again, it seems plausible that for these individuals the social features of the robot were perceived as positive contributors to human-robot interaction.

### Intention to use and level of insight about SAR possibilities

Participants in all three groups reported a higher intention to use the robot in the future than at the present time. This trend was less evident among caregivers, because they took in consideration the decline of cognitive and functional capacities observed over time in persons with dementia. Consequently, they believed that the use of a robot would no longer be possible in the most advanced stages of the disease.

People who felt more concerned by the need of support services, specifically caregivers and persons with MCI, seemed more disposed to discuss practical issues related to the use of the robot, for example, the costs of the service or the need of training and support to use the robot. This is understandable since these individuals had a more pronounced intention to use the system in the present time than HOA. In accordance, they could more easily project themselves acquiring it.

Finally one of the most striking results to emerge from the data was the significant difference observed between current and future intention to use SAR in the MCI and HOA groups. It would seem that these individuals, even those who considered themselves healthy and independent in the present time, were influenced by ageist conceptions and accordingly anticipated a future-self that corresponded to those stereotypes (e.g., being lonely, ill, dependent, or disabled) (Nelson, 2005; McGuire et al., 2008). However, reasons behind these results should be better addressed in future studies.

### Methodological considerations

The diversity and richness of the opinions expressed by participants in this study suggest that mixed-method approaches are particularly well suited to explore potential users' attitudes toward SAR (Dautenhahn, 2007). Questionnaires and focus groups appeared to be complementary approaches. The first one allows the identification of general trends in technology acceptance and the definition of users' profiles whereas the second one is useful to explore more in-depth views. However, combining these two methods poses the question of time and effort required to conduct simultaneously qualitative and quantitative research in terms of sampling and data analysis.

The use of multiple support materials for introducing SAR to participants was effective and could be of general interest for future studies in the field (e.g., live demonstrations, pictures, videos, scenarios). Proof of this is the fact that all participants actively discussed the scenarios for SAR that were provided and developed their own scenarios based on their own experiences. However, preferences regarding robot design and appearance were elicited using exclusively visual material from existing robots. Participants were not given the opportunity to suggest their own design solutions. Including a graphic designer or an artist to sketch the basic outline of ideas suggested by participants in a focus group might be a great brainstorming technique for addressing design issues in this kind of studies. Another drawback of this study is that there was no direct interaction between participants and SAR. Considering that research in the field has shown that familiarity with technology influence users' attitudes toward these systems and increases technology adoption (Young et al., 2009), conducting technology acceptance studies over several sessions and encouraging direct interaction between participants and SAR appears to be an interesting option to study the dynamics of technology acceptance including sequential patterns and attitudes change (Wu et al., 2014).

Some methodological issues limit the findings of this study. First, there is the small size of the sample. A small sample size reduces both, the chance of detecting true effects and the likelihood that a statistically significant result reflects a true effect (Button et al., 2013). For this reason, results from the inferential statistical analyses in this study may have a low predictive value and therefore, should be interpreted with caution. Small sample size also limited the possibility of studying the interaction between individual factors (e.g., education level) and SAR acceptance. Nevertheless, using a small number of subjects appeared to be appropriated to test the hypothesis of different attitudes toward SAR among stakeholder groups and lay the groundwork for future studies in this area.

Some considerations should also be given to the sampling method employed. This study involved exclusively people from the Paris region whose needs and views on SAR may not reflect the perspectives and needs of older people living in different environments (e.g., isolated and rural areas, deprived contexts). Another weakness of this study is that persons with a clinical diagnosis of dementia, one of the prospective primary-users of SAR, were not included in the sample. Although this choice was made to facilitate recruitment and participation in the focus groups, we could have considered adapting user-research methods for involving people with moderate or severe cognitive impairment in the study.

Finally, we summarize here some strategies which may counter some of the problems discussed above that may be useful for future studies on this area: (a) involving a number of participants in each relevant stakeholder group large enough to guarantee the representativeness of the sample and allowing the study of individual factors related SAR acceptance, for instance, performing an a priori power calculation to calculate sample size; (b) better implicating prospective users in SAR design, including persons with dementia, for instance, by organizing co-design workshops; (c) organizing technology acceptance studies over several weeks or months to allow familiarization of participants with SAR, which might show different and more robust behavioral trends.

## Conclusions

It is expected that the field of SAR for dementia care will continue to develop. Still, despite the growing interest in robotics in this context, a specific model of robot acceptance has yet to be developed. This study confirmed that ensuring the design of acceptable and efficient SAR is a complex endeavor. The development of SAR for dementia care requires both, recognizing the needs, expectations and preferences of a range of stakeholders, and better understanding the influence of individual and social factors on technology acceptance. There is no SAR configuration that fits all scenarios. An implication of this is the demand for customizable and highly flexible systems.

Results so far have been encouraging in the sense that they showed that elderly people concerned by cognitive impairment recognize the potential of SAR for supporting health and social care at home. It is true that the current state of the research on SAR does not allow us to conclude that older adults are ready for robots that care for them, but the idea is no longer unimaginable. Nevertheless, many challenges must still be addressed before SAR can be proven reliable, useful, effective, and desirable enough to be introduced as home care assistants.

## Author contributions

MP and MB contributed to the conception and design of the study, data acquisition, analysis and interpretation. MP drafted the article, MB, AR and FJ participated in revising it critically and gave final approval of the version submitted. An earlier version of this study was presented as an oral communication at the Alzheimer's Association 2013 International Conference (AAIC, 2013), Boston (USA) 13-18 July 2013 (Pino et al., 2013).

### Conflict of interest statement

The authors declare that the research was conducted in the absence of any commercial or financial relationships that could be construed as a potential conflict of interest.

## References

[B1] Alaiad A. Zhou L. (2014). The determinants of home healthcare robots adoption: an empirical investigation. Int. J. Med. Inform. 83, 825–840. 10.1016/j.ijmedinf.2014.07.003 25132284

[B2] Alzheimer's Association. (2012). Alzheimer's disease facts and figures. Alzheimers Dement. 8, 131–168. 10.1016/j.jalz.2012.02.001 22404854

[B4] American Psychiatric Association (1994). Diagnostic and Statistical Manual of Mental Disorders. 4th Edn. Washington, DC: American Psychiatric Association.

[B5] Arras K. O. Cerqui D. (2005). Do We Want to Share Our Lives and Bodies with Robots? A 2000-People Survey. Technical Report 0605-001. Autonomous Systems Lab (ASL), Lausanne: Swiss Federal Institute of Technology Lausanne.

[B6] Beer J. B. Prakash A. Mitzner T. L. Rogers W. (2011). Understanding Robot Acceptance. Technical Report HFA-TR-1103. Atlanta, GA Georgia Institute of Technology

[B7] Berger J. Heath C. (2008). Who drives divergence? Identity signaling, outgroup dissimilarity, and the abandonment of cultural tastes. J. Pers. Soc. Psychol. 95, 593–607. 10.1037/0022-3514.95.3.593 18729697

[B8] Blom J. O. Monk A. F. (2003). Theory of personalization of appearance: why users personalize their PCs and mobile phones. J. Hum. Comput. Int. 18, 193–228. 10.1207/S15327051HCI1803_1

[B9] Boissy P. Corriveau H. Michaud F. Labonté D. Royer M.-P. (2007). A qualitative study of in-home robotic telepresence for home care of community-living elderly subjects. J. Telemed. Telecare. 13, 79–84. 10.1258/135763307780096195 17359571

[B10] Broadbent E. Stafford R. MacDonald B. (2009). Acceptance of healthcare robots for the older population: review and future directions. Int. J. Soc. Robot. 1, 319–330. 10.1007/s12369-009-0030-6

[B11] Broadbent E. Kuo I. H. Lee Y. I. Rabindran J. Kerse N. Stafford R.. (2010). Attitudes and reactions to a healthcare robot. Telemed. e-Health 16, 608–613. 10.1089/tmj.2009.0171 20575729

[B12] Broekens J. Heerink M. Rosendal H. (2009). Assistive social robots in elderly care: a review. Gerontechnology 8, 94–103. 10.4017/gt.2009.08.02.002.00

[B13] Button K. S. Ioannidis J. P. Mokrysz C. Nosek B. A. Flint J. Robinson E. S.. (2013). Power failure: why small sample size undermines the reliability of neuroscience. Nat. Rev. Neurosci. 14, 365–376. 10.1038/nrn3475 23571845

[B14] Cui Y. Chipchase J. Ichikawa F. (2007). A cross culture study on phone carrying and physical personalization. Usability and Internationalization. HCI Culture LNCS 4559, 483–492. 10.1007/978-3-540-73287-7_57

[B15] Dautenhahn K. Woods S. Kaouri C. Walters M. Koay K. L. Werry I. (2005). What is a robot companion—friend. assistant or butler?, in Proceedings: International Conference on Intelligent Robots and Systems (IROS 2005) (Edmonton. AB: IEEE Press), 1488–1493.

[B16] Dautenhahn K. (2007). Methodology and themes of human-robot interaction: a growing research field. Int. J. Adv. Robot. Syst. 4, 103–108. 10.5772/5702

[B17] Dedoose (2012). Web Application for Managing, Analyzing, and Presenting Qualitative and Mixed Method Data. Los Angeles, CA: Socio Cultural Research Consultants; LLC.

[B18] DiSalvo C. F. Gemperle F. Forlizzi J. Kiesler S. (2002). All robots are not created equal: the design and perception of humanoid robot heads, in Proceedings 4th Conference on Designing Interactive Systems: Processes, Practices, Methods, and Techniques (London).

[B19] Dupourqué V. (2009). RobuLAB 10, a service robot designed to Aging-in-Place. Gerontechnology 8, 183 10.4017/gt.2009.08.03.009.00

[B20] Feil-Seifer D. Mataric M. J. (2005). Defining socially assistive robotics, in Proceedings of the 2005 IEEE 9th International Conference on Rehabilitation Robotics (Chicago, IL).

[B21] Flandorfer P. (2012). Population ageing and socially assistive robots for elderly persons: the importance of sociodemographic factors for user acceptance. Int. J. Populat. Res. 2012, 1–13. 10.1155/2012/829835

[B22] Folstein M. Folstein S. E. McHugh P. R. (1975). Mini-Mental State” a practical method for grading the cognitive state of patients for the clinician. J. Psychiatr. Res. 12, 189–198. 10.1016/0022-3956(75)90026-6 1202204

[B23] Frennert S. Eftring H. Östlund B. (2013). What older people expect of robots: a mixed methods approach. Soc. Rob. LNCS 8239, 19–29. 10.1007/978-3-319-02675-6_3

[B24] Fujisawa R. Colombo F. (2009). The long-term care workforce: overview and strategies to adapt supply to a growing demand. OECD Health Working Papers. No. 44. OECD Publishing.

[B25] Gross H. M. Schroeter C. Mueller S. Volkhardt M. Einhorn E. Bley A. Langner T. (2011). I'll keep an eye on you: home robot companion for elderly people with cognitive impairment, in Proceedings: 2011 IEEE International Conference on Systems, Man, Cybernetics (SMC) (Anchorage, AK).

[B26] Harmo P. Taipalus T. Knuuttila J. Vallet J. Halme A. (2005). Needs and solutions-home automation and service robots for the elderly and disabled, Proceedings: 2005 IEEE/RSJ International Conference on Intelligent Robots and Systems (Edmonton, AB).

[B27] Hawkey K. Inkpen K. M. Rockwood K. McAllister M. Slonim J. (2005). Requirements Gathering with Alzheimer's Patients and Caregivers, in Proceedings of the 7th International ACM SIGACCESS Conference on Computers and Accessibility (Baltimore, MD).

[B28] Heerink M. Kröse B. Evers V. Wielinga B. (2010). Assessing acceptance of assistive social agent technology by older adults: the almere model. Int. J. Soc. Robot. 2, 361–375. 10.1007/s12369-010-0068-5

[B59] Heerink M. Kröse B. J. A. Evers V. Wielinga B. J. (2006). Studying the acceptance of a robotic agent by elderly users. Int. J. Assist. Robot. Mechatronics 7, 33–43.

[B29] Hirsch T. Forlizzi J. Hyder E. Goetz J. Kurtz C. Stroback J. (2000). The ELDer project: social, emotional, and environmental factors in the design of eldercare technologies, in Proceedings on the 2000 Conference on Universal Usability (Arlington, VA).

[B58] Ho J. Lee C. S. (2011). Factors underlying personalisation adoption: case of mobile telephony. IJSTM 15, 281–297. 10.1504/IJSTM.2011.040380

[B30] Libin A. Cohen-Mansfield J. (2004). Therapeutic robocat for nursing home residents with dementia: preliminary inquiry. Am. J. Alzheimers Dis. Other Demen. 19, 111–116. 10.1177/153331750401900209 15106392 PMC10833783

[B31] MacDorman K. F. Ishiguro H. (2006). The uncanny advantage of using androids in cognitive and social science research. Interact. Stud. 7, 297–337. 10.1075/is.7.3.03mac

[B32] McGuire S. L. Klein D. A. Chen S.-L. (2008). Ageism revisited: a study measuring ageism in East Tennessee, USA. Nurs. Health Sci. 10, 11–16. 10.1111/j.1442-2018.2007.00336.x 18257826

[B33] Meng Q. Lee M. H. (2006). Design issues for assistive robotics for the elderly. Adv. Eng. Inform. 20, 171–186. 10.1016/j.aei.2005.10.003

[B34] Mordoch E. Osterreicher A. Guse L. Roger K. Thompson G. (2013). Use of social commitment robots in the care of elderly people with dementia: a literature review. Maturitas 74, 14–20. 10.1016/j.maturitas.2012.10.015 23177981

[B35] Moyle W. Jones C. Cooke M. O'Dwyer S. Sung B. Drummond S. (2014). Connecting the person with dementia and family: a feasibility study of a telepresence robot. BMC Geriatrics. 14:7. 10.1186/1471-2318-14-7 24456417 PMC3903033

[B36] Nelson T. D. (2005). Ageism: prejudice against our feared future self. J. Soc. Issues 61, 207–221. 10.1111/j.1540-4560.2005.00402.x

[B37] Neven L. (2010). ‘But obviously not for me’: robots, laboratories and the defiant identity of elder test users. Soc. Health Ill. 32, 335–347. 10.1111/j.1467-9566.2009.01218.x 20149151

[B38] Orrell M. Hancock G. A. Liyanage K. C. Woods B. Challis D. Hoe J. (2008). The needs of people with dementia in care homes: the perspectives of users, staff and family caregivers. Int. Psychogeriatr. 20, 941–951. 10.1017/S1041610208007266 18416876

[B39] Portet F. Ousset P. J. Visser P. J. Frisoni G. B. Nobili F. Scheltens P.. (2006). Mild cognitive impairment (MCI) in medical practice: a critical review of the concept and new diagnostic procedure. Report of the MCI Working Group of the European Consortium on Alzheimer's Disease. J. Neurol. Neurosurg. Psychiatry 77, 714–718. 10.1136/jnnp.2005.085332 16549412 PMC2077456

[B40] Pino M. Boulay M. Rigaud A.-S. (2013). Acceptance of social assistive robots to support older adults with cognitive impairment and their caregivers. Alzheimers Dement. 9, P342 10.1016/j.jalz.2013.04.205

[B41] R Development Core Team (2011). R: A Language and Environment for Statistical Computing. Vienna: R Foundation for Statistical Computing Available online at: http://www.r-project.org/

[B42] Rabbitt S. M. Kazdin A. E. Scassellati B. (2015). Integrating socially assistive robotics into mental healthcare interventions: applications and recommendations for expanded use. Clin. Psychol. Rev. 35, 35–46. 10.1016/j.cpr.2014.07.001 25462112

[B43] Rich C. Sidner C. L. (2009). Robots and avatars as hosts, advisors, companions, and jesters. AI Magazine 30, 29–41 (Accessed, October 12 2013).

[B44] Robinson H. MacDonald B. A. Kerse N. Broadbent E. (2013). Suitability of healthcare robots for a dementia unit and suggested improvements. JAMDA 14, 34–40. 10.1016/j.jamda.2012.09.006 23098418

[B45] Scopelliti M. Giuliani M. V. Fornara F. (2005). Robots in a domestic setting: a psychological approach. UAIS 4, 146–155. 10.1007/s10209-005-0118-1

[B46] Sharkey A. Sharkey N. (2010). Granny and the robots: ethical issues in robot care for the elderly. Ethics Inform. Technol. 14, 27–40. 10.1007/s10676-010-9234-6

[B47] Shibata T. Wada K. (2010). Robot therapy: a new approach for mental healthcare of the elderly-a mini-review. Gerontology 57, 378–386. 10.1159/000319015 20639620

[B48] Sparrow R. Sparrow L. (2006). In the hands of machines? The future of aged care. Minds Machines 16, 141–161. 10.1007/s11023-006-9030-6

[B49] Strauss A. Corbin J. M. (1998). Basics of Qualitative Research: Techniques and Procedures for Developing Grounded Theory, 2nd Edn. Thousand Oaks, CA: SAGE Publications.

[B50] Sung J. Y. Grinter R. E. Christensen H. I. (2009). Pimp my Roomba: designing for personalization, in Proceedings of the 27th International Conference on Human Factors in Computing Systems (Boston, MA).

[B51] Topo P. Saarikalle K. Begley E. Cahill S. Holthe T. Macijauskiene J. (2007). I don't know about the past or the future, but today it's Friday”—Evaluation of a time aid for people with dementia. Technol. Disabil. 19, 121–131.

[B52] Topo P. (2009). Technology studies to meet the needs of people with dementia and their caregivers: a literature review. J. Appl. Gerontol. 28, 5–37. 10.1177/073346480832401917869590

[B53] van der Roest H. G. Meiland F. J. van Hout H. P. Jonker C. Droes R. M. (2008). Validity and reliability of the Dutch version of the Camberwell assessment of need for the elderly in community-dwelling people with dementia. Int. Psychogeriatr. 20, 1273–1290. 10.1017/S1041610208007400 18554426

[B60] Venkatesh V. Morris M. G. Davis G. B. Davis F. D. (2003). User acceptance of information technology: toward a unified view. MIS Q. 27, 425–478. 10.2307/30036540

[B54] Walters M. L. Koay K. L. Syrdal D. S. Dautenhahn K. Te Boekhorst R. (2009). Preferences and perceptions of robot appearance and embodiment in human-robot interaction trials, in Proceedings: Symposium at the AISB09 Convention (Edinburgh).

[B55] Wu Y.-H. Fassert C. Rigaud A.-S. (2012). Designing robots for the elderly: appearance issue and beyond. Arch. Gerontol. Geriat. 54, 121–126. 10.1016/j.archger.2011.02.003 21349593

[B56] Wu Y.-H. Wrobel J. Cornuet M. Kerhervé H. Damnée S. Rigaud A.-S. (2014). Acceptance of an assistive robot in older adults: a mixed-method study of human-robot interaction over a 1-month period in the Living Lab setting. Clin. Interv. Aging 9, 801–811. 10.2147/CIA.S56435 24855349 PMC4020879

[B57] Young J. E. Hawkins R. Sharlin E. Igarashi T. (2009). Toward acceptable domestic robots: applying insights from social psychology. Int. J. Soc. Robot. 1, 95–108. 10.1007/s12369-008-0006-y

