# Supplementary material for: “Are we ready for robots that care for us?” Attitudes and opinions of older adults toward socially assistive robots
Source: Front Aging Neurosci. 2015 Jul 23;7:141. doi: 10.3389/fnagi.2015.00141 (PMC4512026; doi:10.3389/fnagi.2015.00141)
Supplement: Supplementary file 1 [file Presentation1.PDF]

Appendix 1. Questionnaire "Social Assistive Robots"

Part A

Socio-demographic data

Age________

Gender: ☐ F ☐ M

Education Level ☐ Primary ☐ Secondary ☐ Higher (University)

Employment status ☐ Active ☐ Retired

Health status

What are the main difficulties you experience in your daily life? :

☐ memory problems ☐ finding words or names ☐ falling or being afraid to fall

☐ moving around ☐ administrative paperwork and personal finances

☐ sadness, depression ☐ anxiety ☐ apathy

☐ solitude or isolation ☐ chronic diseases (diabetes ; hypertension, etc.)

☐ pain ☐ other __________________

Technology experience

Amongst the following technologies which of them do you currently use? (at least once a week)

☐ radio ☐ television set ☐ landline telephone ☐ cell phone ☐ answering machine

☐ DVD player ☐ camera ☐ digital music player ☐ computer ☐ Internet

☐ tablet ☐ micro wave ☐ dishwasher ☐ e-mail ☐ Automatic ticket vending machine

What interest level do you have for new technologies in general?

☐ very interested ☐ fairly interested ☐ not very interested ☐ not interested at all

How do you normally react to a new technology-related service or product?

☐ always adopt ☐ adopts most of the time

☐ adopts occasionally ☐ almost never adopts

Part B

General appreciation of Social Assistive Robots

Please indicate your three (3) favorite personal robots in terms of physical appearance (Pictures and description of robots were given in a supplementary booklet)

☐ machine-like robots ☐ mechanical human-like robots ☐ human-like robots

☐ animal-like robots ☐ mechanical animal-like robots ☐ androids

How important is that the robot has a head with realistic human traits?

☐ very important ☐ important ☐ not very important ☐ not important at all

How important is that the robot is capable of expressing emotions?

☐ very important ☐ important ☐ not very important ☐ not important at all

Which are the three (3) applications that you would prefer for a robot. Please write your response in order of importance:

(1)________________ **(2)**________________ **(3)**________________

A robot would be useful to me in my current situation

☐ absolutely ☐ fairly yes ☐ fairly no ☐ not at all Why?__________________

I plan to use a robot in your home at the present time

☐ absolutely ☐ fairly yes ☐ fairly no ☐ not at all Why?__________________

I plan to use a robot in your home in the future

☐ absolutely ☐ fairly yes ☐ fairly no ☐ not at all Why?__________________
