# Supplementary material for: “Are we ready for robots that care for us?” Attitudes and opinions of older adults toward socially assistive robots
Source: Front Aging Neurosci. 2015 Jul 23;7:141. doi: 10.3389/fnagi.2015.00141 (PMC4512026; doi:10.3389/fnagi.2015.00141)
Supplement: Supplementary file 2 [file Presentation2.PDF]

Appendix 2. Description of SAR (focus group booklet)

Name Producer, (country)	Picture	Type of robot	Description
**RobuLAB 10**<br>Robosoft<br>(France)	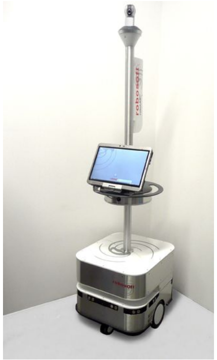	Machine-like	- Mobile platform<br>- Embarks a group of sensors and cameras for autonomous navigation,<br>- Input devices include speech control and touch-screen<br>- System programmed to provide cognitive and social support through different applications (e.g., task reminder, cognitive training, navigation support, communication tools)
**Kompaï**<br>Robosoft<br>(France)	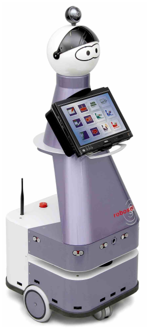	Mechanical human-like	- Same configuration as Robulab but with a different appearance (human-like head)
**Pearl**<br>Carnegie Mellon University<br>(USA)	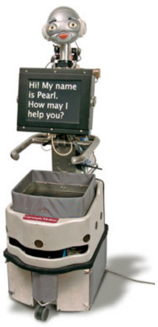	Mechanical human-like	- Mobile platform<br>- Designed to help older adults to navigate through a nursing facility<br>- Provides advice, cognitive support, functional assistance
**Mamoru**<br>University of Tokyo<br>Toyota and Fujitsu<br>(Japan)	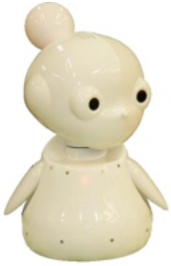	Mechanical human-like	- Desktop elder-care robot<br>- Designed to provide prompts and reminders (e.g., Item location, medication intake)<br>- Uses a wide-angle camera to detect objects regardless of rotation, scale, or lighting conditions
**EVE**<br>PIXAR, DISNEY studios<br>(USA)	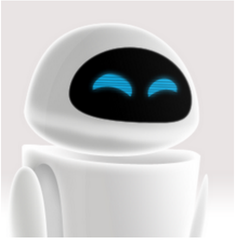	Mechanical human-like	- Animated female robot from the film Wall-E<br>- Mix between human and machine features<br>- Emotions represented through facial expressions and voice

Name Producer, (country)	Picture	Type of robot	Description
**Telenoïd** Osaka University, ATR (Japan)	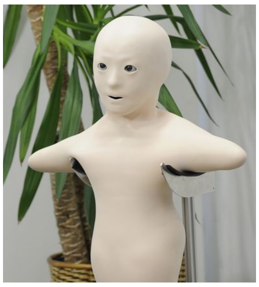	Human-like	- Humanoid robot with minimal human appearance<br>- High quality silicon human mimicking skin<br>- Designed to be a communication device, with applications in remote work, education, and elderly care<br>- Allows distant transmission of “human presence” (e.g., voice, face and head movements)
**Nexi** MIT Media Lab (USA)	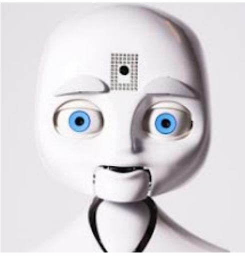	Human-like	- Mobile manipulator robot capable of social expression<br>- Applications for personal robots and human-robot teamwork<br>- Hands for manipulating objects, eyes (video cameras), ears (an array of microphones), 3-D infrared camera and laser rangefinder to support real-time tracking of objects, people, voices, and indoor navigation
**Geminoid F** Osaka University, ATR (Japan)	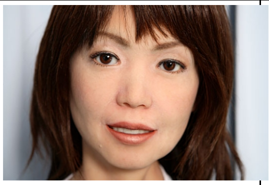	Android	- Android that works as a substitute for clerks, when typical responses are required<br>- Consists in three elements: a robot, a central controlling server, and a teleoperation interface
**iCat** Philips Electronics (The Netherlands)	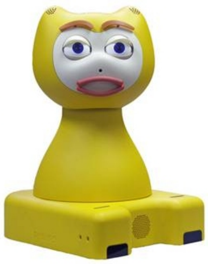	Mechanical animal-like	- Cat-like appearance<br>- Face expressing emotions<br>- Aimed at functional assistance<br>-Strongly relates to social interaction
**Paro** Intelligent Systems Research Institute (ISRI) (Japan)	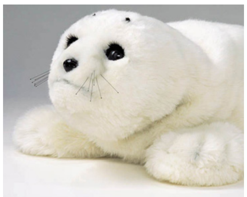	Animal-like	- Soft seal robot developed for robot therapy in older adults with cognitive impairment, and other populations with disabilities<br>- Programmable behavior as well as a set of sensors (touch sensor, infrared sensor, stereoscopic vision and hearing).<br>- Actuators include eyelids, upper body motors, front paw and hind limb motors.
